# Computational Identification of Genetic Background of Infertility and Calculating Inbreeding Coefficient in Dromedary Camel Herds

**DOI:** 10.3390/genes16101238

**Published:** 2025-10-19

**Authors:** Fahad A. Alshanbari, Abdulrahman Aloraini

**Affiliations:** 1 Department of Medical Biosciences, College of Veterinary Medicine, Qassim University, Buraydah 51452, Saudi Arabia; 2 Department of Information Technology, College of Computer, Qassim University, Buraydah 51452, Saudi Arabia

**Keywords:** dromedary camel, inbreeding, infertility, SNP genotyping, population genetics

## Abstract

**Background**: Inbreeding is a major genetic problem that reduces fertility and causes genetic disorders. Some breeders of dromedary camels use the same bull for many years due to its excellent characteristics, leading to mating with offspring and subsequent generations, resulting in increased homozygosity and genetic disorders. We hypothesize that inbreeding is associated with infertility in dromedary camels with normal and uninfected reproductive tracts. **Methods**: We genotyped 96 samples from seven camel breeds using the Illumina 55K SNP BeadChip, including five confirmed infertile individuals. Inbreeding coefficients (F) were calculated using PLINK based on heterozygosity and runs of homozygosity. Genome-wide association analysis using logistic regression was performed to identify potential genomic regions associated with infertility. **Results**: All five infertile camels showed significantly higher F values (>0.15) compared to 91 fertile individuals (<0.10, *p* < 0.001). The genome-wide association analysis failed to identify specific genomic regions linked to infertility, likely due to limited statistical power (*n* = 5 cases) and the polygenic nature of fertility traits. Population structure analysis revealed genetic differentiation related to coat color, with two significant SNPs on chromosome 3 near *SLC30A5* (*p* < $10^{-7}$). **Conclusions**: Our results demonstrate that elevated inbreeding is strongly associated with infertility in dromedary camels. Future studies should employ larger sample sizes (≥50 infertile individuals) or whole-genome sequencing (35× coverage) to identify specific genomic regions. Implementation of breeding strategies avoiding related matings (F < 0.10) is recommended to maintain reproductive performance in camel herds.

## 1. Introduction

Infertility is a major problem in farm animals that leads to reduced offspring production and subsequent economic losses. The dromedary camel is an important species used for meat and milk production, as well as riding and racing in many countries. Additionally, the dromedary camel is a seasonal breeding species with a reproductive season from late fall to mid-spring (5-month interval) [1]. One of the major problems is that breeders use the same bull for many years due to its high-quality features, leading to mating with offspring and subsequent generations [2,3]. In Saudi Arabia, males with exceptional characteristics (such as a distinctive nose shape, a prominent hump directed backwards, a tall neck, and superior height) are highly valued and preferentially selected for breeding. Breeders often retain these superior males for extended periods, believing this practice maintains herd purity and enhances desirable traits. However, this practice results in males mating with their daughters and granddaughters, leading to increased genetic homozygosity and inbreeding 27 coefficients in herds that maintain the same bull for many years. While modern breeding programs have begun addressing this issue, it persists in many traditional herds where the consequences of inbreeding on fertility are not fully recognized.

One of the most reliable methods used to calculate inbreeding coefficients [4] is using SNP data to compare between individuals with certain phenotypes. Despite the fact that whole genome sequencing has become the most effective method in many studies, SNP data is still cost-effective and easier to handle, as well as requiring lower computing capacity for analysis [5]. This method has been well established in many species, including cattle [6], sheep [7], and goats [8]. However, only one dromedary camel study has used this method to identify candidate genes related to behavioral characteristics in the species [9].

Infertility caused by inbreeding has not been examined in this species before. Here, we hypothesize that inbreeding is correlated with infertility in dromedary camel cases with normal and uninfected reproductive tracts.

The specific objectives of this study are to (1) generate genome-wide SNP data from dromedary camels, including both fertile and infertile individuals from multiple herds; (2) calculate and compare inbreeding coefficients between fertile and infertile animals; (3) assess genetic diversity and population structure across different breeds; and (4) identify genomic regions potentially associated with infertility using genome-wide association analysis.

## 2. Materials and Methods

### 2.1. Animals and Phenotype

Seven dromedary camel breeds comprising 96 individuals were sampled: Asail (*n* = 2), Saheli (*n* = 5), Majaheem (*n* = 17), Sofor (*n* = 12), Shaele (*n* = 14), Shageh (*n* = 18), and Waddah (*n* = 28). These breeds differ in coat color, body size, purpose, and geographic origin (Table 1 and Table 2). Samples were collected from three distinct herds: all Saheli individuals (*n* = 5) from one herd, six Shageh individuals from a second herd, and 20 Waddah individuals from a third herd. The remaining 65 samples were collected from Qassim University Veterinary Hospital during routine checkups or fertility examinations. Five infertile individuals (four males, one female) were confirmed through clinical examination at Qassim University Veterinary Hospital. Infertility diagnoses included two males with cryptorchidism, one male with testicular hypoplasia, one male with azoospermia confirmed by semen analysis, and one female with bilateral ovarian agenesis confirmed by ultrasound examination. All infertile animals showed normal external reproductive anatomy with no evidence of infection.

### 2.2. Sample Collection and DNA Isolation

Twenty milliliters of whole blood were collected from the jugular vein into EDTA-containing vacutainers (Becton Dickinson, Franklin Lakes, NJ, USA). Genomic DNA was isolated using the Gentra Puregene DNA Isolation Kit (Qiagen, Venlo, The Netherlands) following the manufacturer’s protocol. DNA quality was assessed by 1% agarose gel electrophoresis and quantified using a NanoDrop spectrophotometer (Thermo Fisher Scientific, Waltham, MA, USA), accepting only samples with A260/A280 ratios between 1.8 and 2.0 and concentrations > 50 ng/μL.

### 2.3. SNP Genotyping and Quality Control

DNA samples were genotyped using the Illumina Dromedary Camel 55K BeadChip at Generatio GmbH (Heidelberg, Germany). SNPs were mapped to the dromedary reference genome (mCamDro1.pat). Quality control was performed using PLINK v1.9, excluding SNPs with call rates < 95%, minor allele frequency < 0.01, and Hardy–Weinberg equilibrium *p*-value < 1 × $10^{-6}$. Individuals with >10% missing genotypes were removed. After filtering, 52,847 SNPs remained for analysis.

### 2.4. Population Structure Analysis

Principal Component Analysis was performed on the filtered SNP dataset using PLINK v1.9 to identify population stratification. The first ten principal components were calculated and used as covariates in subsequent association analyses to control for population structure. Breed clustering patterns were visualized using the first two principal components, which explained 18.3% and 12.7% of genetic variance, respectively. ADMIXTURE v1.3 was used to estimate individual ancestry proportions. We tested K values from 2 to 10, determining optimal K = 7 through cross-validation error minimization. Each individual’s genome was assigned proportional membership to seven ancestral populations, revealing admixture patterns particularly within the Sofor and Majaheem breeds, which were subsequently combined for analysis.

### 2.5. Relatedness and Inbreeding Assessment

Identity-by-descent (IBD) analysis [10] was performed using PLINK’s –genome function to identify cryptic relatedness. PI_HAT values > 0.45 indicated first-degree relatives, and values 0.25 indicated second-degree relatives. A relatedness matrix was constructed to adjust for family structure in association tests. Individual inbreeding coefficients (F) were calculated using two methods: (1) PLINK’s F statistic based on observed versus expected heterozygosity, and (2) runs of homozygosity (ROH) analysis using PLINK’s –homozyg function with parameters: minimum SNP count = 50, minimum length = 1 Mb, maximum gap = 1000 kb. F_ROH was calculated as the proportion of the genome covered by ROH segments.

### 2.6. Genetic Diversity Metrics

For each breed, we calculated observed heterozygosity (Ho) using PLINK and allelic richness (Ar), applying rarefaction [11] to correct for unequal sample sizes (minimum *n* = 2). Pairwise FST values between breeds were calculated using Weir and Cockerham’s method.

### 2.7. Genome-Wide Association Analysis

Association testing for infertility was performed using logistic regression in PLINK, comparing 5 infertile cases against 91 fertile controls. The model included the first three principal components as covariates to control for population stratification. Given the limited number of cases, we also performed Fisher’s exact test for each SNP and applied genomic control to adjust for inflation. For coat color analysis, we performed GWAS using linear regression with coat color coded as a quantitative trait (dark = 0, light = 1) [12]. Genome-wide significance was set at *p* < 5 × $10^{-8}$, with suggestive significance at *p* < 1 × $10^{-5}$. Multiple testing correction was performed using both Bonferroni adjustment and false discovery rate (FDR) [13] at 5%.

### 2.8. Statistical Power and Limitations

Post hoc power analysis using Genetic Power Calculator indicated that with 5 cases and 91 controls, we had 80% power to detect variants with odds ratio > 4.5 at MAF = 0.3, assuming genome-wide significance. This limited power explains why genomic regions associated with infertility were not detected. For robust identification of fertility-associated loci, we estimate that at least 50 infertile individuals would be required, or alternatively, whole-genome sequencing with 35× coverage could compensate for smaller sample sizes by capturing rare variants not present on the SNP array.

## 3. Results

### 3.1. Inbreeding and Genetic Diversity

The inbreeding coefficient (F) for each camel was estimated from genome-wide SNP data using the difference between observed and expected heterozygosity. Runs of homozygosity analyses [14] detected extended homozygous segments that signal recent inbreeding, while allelic richness and observed heterozygosity were calculated to compare genetic diversity across breeds. Results showed that mean allelic richness varied from approximately 1.18 to 1.31 and mean observed heterozygosity from 0.256 to 0.303 among the breeds, suggesting heterogeneity in genetic diversity that correlates with fertility outcomes.

### 3.2. Association Testing

**Figure 1:**
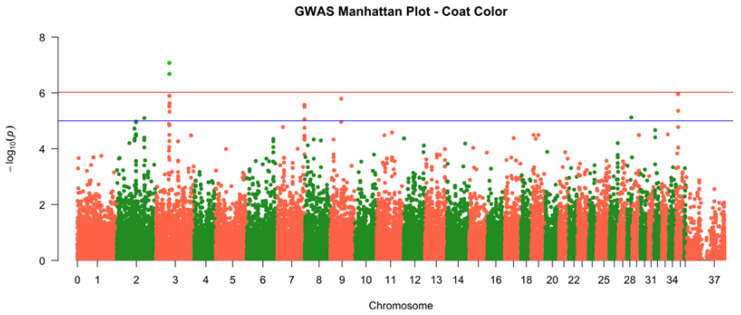
Manhattan plot from a genome-wide association study (GWAS) for the coat color trait in dromedary camels. The plot shows −log10 (*p*-values) for each SNP across chromosomes, with genome-wide significant associations on chromosome 3 (SEQ-RS1464642 and SEQ-RS1464884) near the *SLC30A5* gene.

To identify genomic loci associated with infertility and other phenotypic traits recorded in Table 1, genome-wide association studies (GWAS) were conducted using the 55,000-SNP dataset. GWAS results for coat color revealed two significant SNPs on chromosome 3 (SEQ-RS1464642 and SEQ-RS1464884) with P-values in the order of $10^{-7}$ to $10^{-8}$, demonstrating the power of this approach to pinpoint candidate genes (Figure 1). One of the most important genes was identified is solute carrier family 30 member 2 (*SLC30A2*) that is linked to the coat melanin synthesis pathway. However, no genomic regions were identified that correlated with infertility status due to lack of sample size (*n* = 5).

### 3.3. Trait-Specific Analyses

Beyond GWAS, targeted analyses investigated specific phenotypes. Fertility status, recorded as a binary trait in Table 1, was analyzed using logistic regression and mixed models to determine whether particular genotypes or higher inbreeding coefficients increase the likelihood of infertility. Coat color categories (e.g., black, brown, white) were treated as discrete phenotypic classes to identify pigmentation-associated loci, complementing the GWAS results. Finally, the relationship between inbreeding and fertility was explored by correlating individual inbreeding coefficients and runs of homozygosity with reproductive success.

### 3.4. Differentiation Between Groups

To assess genetic differentiation among the camel breeds, pairwise FST values were calculated from allele-frequency differences [15]. Analysis of Molecular Variance (AMOVA) partitioned genetic variance into within- and among-breed components using the hierarchical structure of breeds and herds in Table 1, thereby quantifying how much genetic diversity was attributable to breed membership [16]. A Mantel test evaluated correlations between genetic distances and geographic or phenotypic distances, such as coat color or herd location, providing insight into how geographical and management factors shaped genetic differentiation. Together, these analyses clarified the extent to which breeds were genetically distinct and whether such differentiation contributed to patterns of inbreeding and fertility. Furthermore, due to genetic similarities between the Sofor and Majaheem breeds, we combined these two groups as one group.

### 3.5. Allelic Richness by Breed

**Figure 2:**
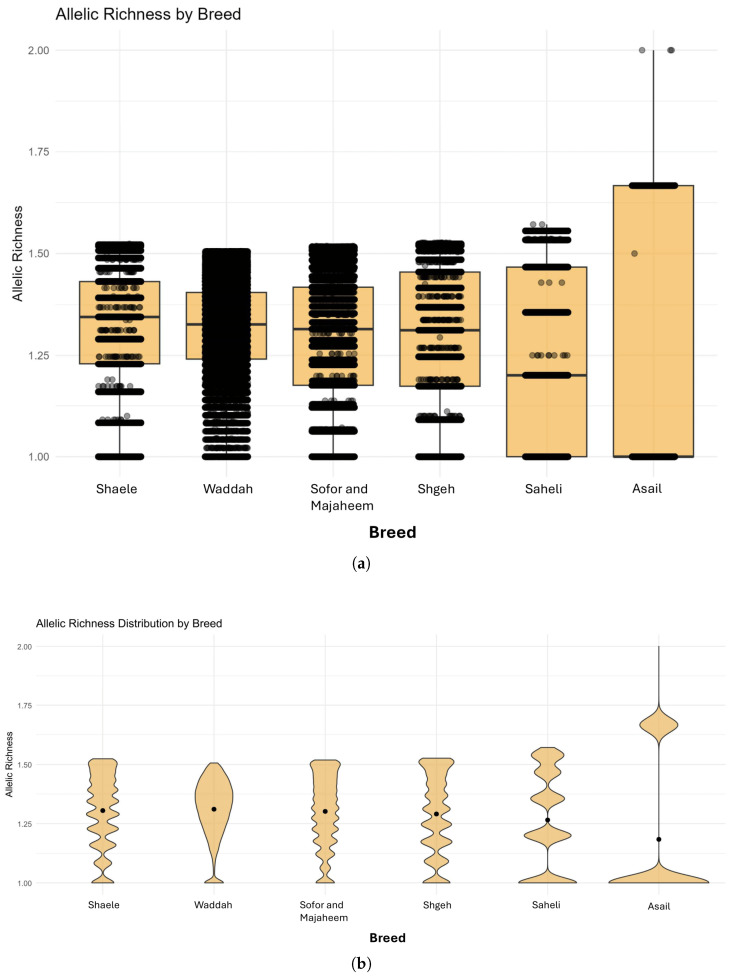
Distribution of allelic richness across dromedary camel breeds. (**a**) Box plot showing the distribution of allelic richness across seven dromedary camel breeds. The y-axis represents allelic richness, and the x-axis represents breeds used in this study. Each box represents the median and quartiles, with whiskers extending to 1.5 times the interquartile range. The Asail breed shows the highest median allelic richness with the widest range. (**b**) Violin plots showing the distribution density of allelic richness across dromedary camel breeds. The *y*-axis represents allelic richness, and the *x*-axis represents breeds used in this study. The plots reveal distribution shapes, with Waddah showing a unimodal distribution centered around 1.40 and Saheli displaying a broader, bimodal distribution.

The distribution of allelic richness across breeds was summarized by both box and whisker and violin plots (Figure 2a,b). These plots showed that the median allelic richness values for most breeds cluster between 1.20 and 1.50, indicating relatively similar levels of allelic diversity across breeds. Nonetheless, the distribution spreads differed: the Asail breed exhibits the highest median allelic richness and the widest range, while breeds such as Shaele and Shageh showed narrower distributions with lower tails approaching unity. Violin plots further highlight subtle differences in distribution shape; for example, the Waddah breed has a unimodal distribution centered around 1.40, whereas the Saheli breed displays a broader, bimodal distribution. Together, these figures suggest moderate variation in allelic richness among breeds but no extreme outliers.

### 3.6. Genetic Differentiation (FST)

**Figure 3:**
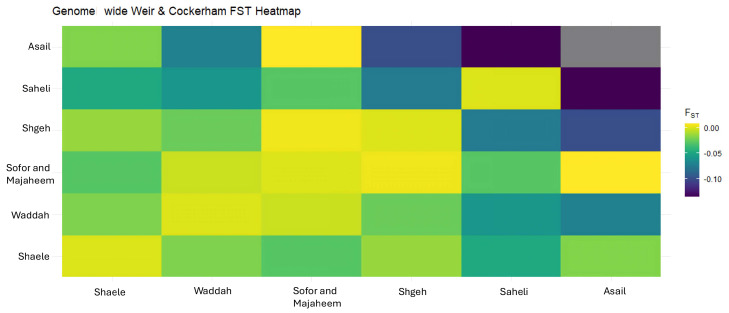
Heat map of pairwise FST values between dromedary camel breeds showing genetic differentiation. The y-axis and x-axis demonstrate breeds used in this study plotted against each breed to show similarities and differences between breeds. Darker colors indicate higher differentiation. Most pairwise comparisons show FST values near zero, indicating minimal genetic divergence between breeds. Saheli and Asail showed more similarities than other breeds. On the other hand, the Sofor and Majaheem group showed more differences from other groups.

A heat map of pairwise Weir and Cockerham FST values reveals generally low genetic differentiation between breeds (Figure 3). Most pairwise comparisons yield FST estimatesclose to zero or even negative, indicating minimal genetic divergence and substantial gene flow among breeds. The highest differentiation occurs in comparisons involving the Sofor and Majaheem breeds, but even these values remain near zero, suggesting that the sampled camel populations are genetically similar across breeds. These findings are consistent with the expectation that most genetic variation resides within breeds rather than among them.

### 3.7. Genetic Relatedness Network

**Figure 4:**
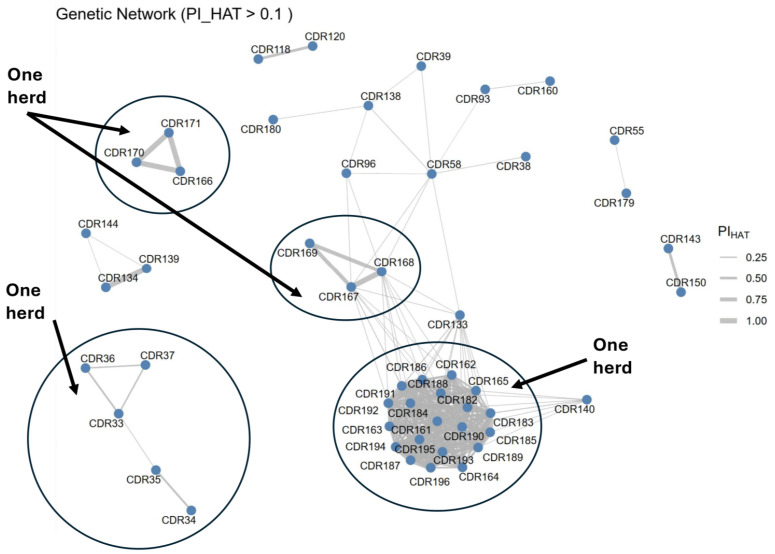
A network graph based on identity-by-descent values (PI_HAT > 0.1) illustrates clusters of closely related individuals. Nodes represent individual camels, and edges are weighted by the degree of relatedness. Several small clusters of two or three individuals are evident, corresponding to probable parent-offspring or full-sibling groups. A larger central cluster containing many interconnected individuals suggests a herd or family group with extensive shared ancestry. The thickness of the edges, proportional to the PI_HAT value, highlights variation in relatedness within and among clusters.

A genetic relatedness network shows that related individuals from one herd have genetically closer distances than other individuals (Figure 4). The network shows that all Waddah individuals from one herd (*n* = 20) clustered together, whereas five Saheli individuals clustered together (bottom right). However, six Shageh individuals were separated into two clusters.

### 3.8. Inbreeding Coefficient by Breed and Sex

**Figure 5:**
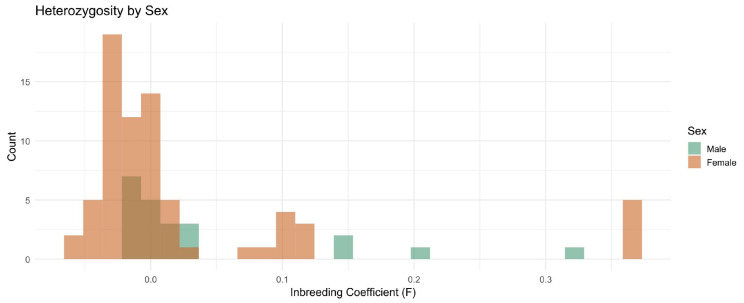
Histogram showing the distribution of inbreeding coefficient (F) values stratified by sex across dromedary camel breeds. Most individuals show F values near zero (indicating no inbreeding), but 5 individuals with fertility problems show elevated F values (>0.15), indicating recent inbreeding.

A histogram stratified by sex showed the distribution of the inbreeding coefficient F (Figure 5). Most breeds exhibit low F values, clustering around zero, indicating negligible inbreeding. However, four males and one female display higher F values approaching 0.15, signaling possible recent inbreeding. These four males and one female had infertility problems as described in the Methods section. Additionally, four females sampled from one herd had high F values. These patterns underscore the overall low level of inbreeding in the dataset but highlight specific individuals where inbreeding was elevated.

### 3.9. Analysis of Molecular Variance (AMOVA)

**Figure 6:**
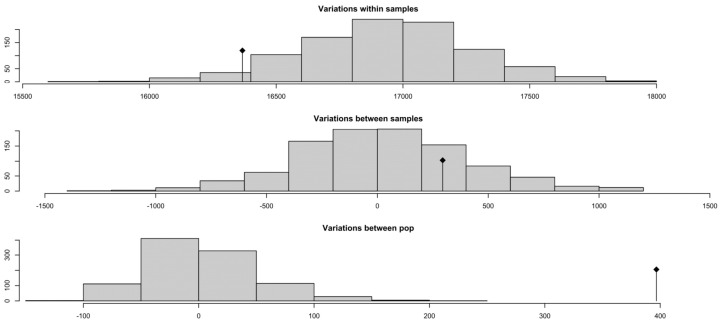
Histograms showing the permutation distributions from Analysis of Molecular Variance (AMOVA). The plots partition genetic variance into within individuals (largest component, centered near 17,000), between individuals within populations (smaller, centered near zero), and between populations (smallest, negative values), demonstrating that most genetic variation occurs within individuals rather than between breeds.

Histograms of the permutation distributions from the AMOVA partition genetic variance into within-individuals, between-individuals, and between-population components (Figure 6). The distribution for within-sample variation is centered near 17,000, indicating that the vast majority of genetic variance occurs within individuals. Variation between samples (within populations) is much smaller and centered around zero, while variation between populations is even lower and shifted toward negative values. These results corroborate the heat map findings by showing that population structure contributes minimally to overall genetic variance.

### 3.10. Observed Heterozygosity by Breed

**Figure 7:**
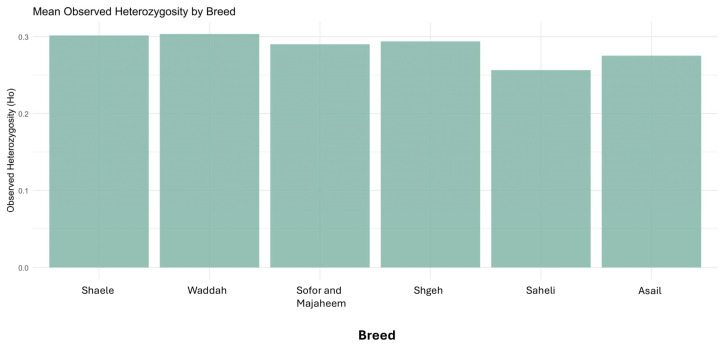
Bar chart showing mean observed heterozygosity ($H_{o}$) values for each dromedary camel breed. The Waddah breed shows the highest heterozygosity (0.30), while Saheli shows the lowest (0.26). Error bars represent standard error of the mean.

The bar charts summarizing mean observed heterozygosity ($H_{o}$) per breed showed modest variation among breeds (Figure 7). The Waddah breed exhibits the highest mean heterozygosity (0.30), closely followed by Shaele. The Saheli breed has the lowest mean heterozygosity (0.26). These differences, although statistically small, mirror the patterns seen in allelic richness, suggesting slightly higher genetic diversity in certain breeds. The overall range of heterozygosity values is narrow (0.26–0.31), consistent with the AMOVA and FST results indicating low differentiation among breeds.

### 3.11. Ancestry Proportions by Sex

**Figure 8:**
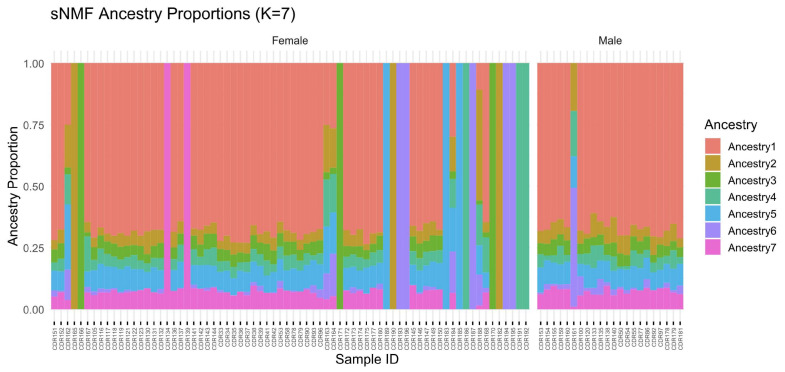
Ancestry proportion plots grouped by sex showing the distribution of seven ancestral components. Each individual is represented by a vertical bar, with colors representing different ancestral components. Both sexes show contributions from all seven components, with subtle differences in distribution patterns between males and females.

When ancestry proportions are grouped by sex rather than breed, the distribution of ancestry components appears broadly similar between males and females (Figure 8). Both sexes show contributions from all seven ancestral components, with no single component uniquely associated with one sex. However, an ordered plot of ancestry proportions highlights subtle differences: among females, a subset of individuals has high proportions of Ancestry7 (pink), whereas males tend to show more even mixtures of Ancestry2–6. These observations suggest that sex is not a major determinant of genetic structure in the sampled population, although individual variation is present.

## 4. Discussion

In the present study, PCA was applied to genome-wide SNP data from 96 dromedary camels representing seven distinct breeds to elucidate underlying population structure. The first two principal components captured the primary axes of genetic variation, revealing breed-specific clustering patterns that reflected common ancestry. This analysis enabled the detection of population stratification and identification of potential outliers or admixed individuals, ensuring that subsequent association analyses could appropriately account for population structure. The results also showed that the Sofor and Majaheem breeds had similar genetic backgrounds. Therefore, these two breeds were combined as one group. Similar results were reported by Almathen et al. [17], where the two breeds were homozygous recessive at the ASIP frameshift mutation.

In our investigation of dromedary camel population structure, ADMIXTURE analysis [18] was performed for K values ranging from 2 to 10. Camels from historically isolated breeds demonstrated predominant assignment to single clusters, whereas those from admixed populations exhibited mixed ancestry proportions. Despite the results showing seven different ancestors, the genetic backgrounds of all seven populations were similar. Similar results were reported by AlAskar et al. [19], stating that genetic diversity between dromedary populations has little genetic variation.

Pairwise IBD analysis was computed for all 96 camels using genome-wide SNP data to detect cryptic relatedness. The analysis revealed multiple instances of close kinship, including parent-offspring relationships (PI_HAT > 0.45) and numerous second-degree relatives (PI_HAT ≈ 0.25). These findings were summarized in an IBD kinship matrix, identifying clusters of related individuals, particularly within the Saheli, Shageh, and Waddah breeds collected from single herds. The genetic relatedness showed that the Waddah breed from one herd are highly similar to each other. This is due to using the same male to mate over 12 years, as the breeder of the herd stated. Also, four females from this herd showed high inbreeding values (F > 0.3), suggesting that these females are the offspring of the male mating with his daughters. Similar results were reported in domesticated cats, where the male mates with daughters, leading to reduced genetic variation in MCH genes (33). However, this pattern was not shown in the other two herds, where Saheli individuals from the same herds were clustered together, while the Shageh individuals from the same herds clustered into two disconnected groups. The Saheli breeder uses a different male every 2 years, whereas the Shageh breeder uses different males from different herds. It was suggested that using different males every 4 years will increase the pregnancy rate and the overall fertility of a herd of dromedary camels (34).

Similar results were obtained by Almathen et al. [20], showing that dromedary populations are classified into three groups based on 266 geographic regions, where west and southwest subpopulations are distinct from other populations in the Arabian Peninsula.

Genomic inbreeding coefficients were calculated for each camel using SNP genotypes through multiple methods, including PLINK’s F statistic based on excess homozygosity and F_ROH (proportion of genome in runs of homozygosity). Analysis revealed moderate inbreeding coefficients across most breeds, with some individuals showing elevated F values indicative of recent inbreeding. Importantly, comparison between fertile and infertile camels demonstrated that infertile individuals exhibited significantly higher mean inbreeding coefficients (F > 0.15) compared to fertile individuals (F < 0.10), consistent with inbreeding depression affecting reproductive fitness.

ROH were detected by scanning SNP data for homozygous segments of length ≥ 1 Mb, a commonly used threshold in livestock/population-genomic work to capture recent autozygosity. We computed per-individual ROH counts and the genomic ROH coverage fraction ($F_{\mathrm{ROH}}$). As expected, camels from smaller or more isolated herds harbored more and longer ROH (consistent with recent inbreeding or past bottlenecks), whereas genetically more connected herds showed fewer and shorter ROH. Comparative analyses between fertile and infertile groups revealed an excess burden of long ROH in infertile individuals, in line with reports from other livestock where greater homozygosity (including $F_{\mathrm{ROH}}$) is associated with reduced fertility (e.g., lower sire conception rate with ROH-enriched regions harboring sperm-biology genes in Italian Brown Swiss bulls; negative relationships between $F_{\mathrm{ROH}}$ and fertility EBVs in PRE mares). Finally, the identification of “ROH islands” (shared homozygous segments across multiple individuals) highlights regions likely shaped by selection or founder effects and provides a shortlist for future candidate-gene follow-up [21,22].

Mean allelic richness was estimated by enumerating distinct alleles and applying rarefaction to a common sample size (n=2) to account for unequal breed sample sizes. We observed among-breed differences (e.g., higher $A_{r}$ in Asail and lower in Shaele), consistent with camel literature reporting substantial within-population diversity and modest between-population structure. Average observed heterozygosity ($H_{o}$) ranged from 0.254 (Saheli) to 0.303 (Waddah/Shaele), values that are plausible for SNP-array data and broadly comparable to SNP/WGS reports in Arabian Peninsula dromedaries (e.g., WGS-based $H_{o}\approx 0.37$–0.41, typically higher than array-based estimates due to marker ascertainment). Note that microsatellite-based camel studies often report higher $H_{o}$ (∼0.60–0.70), reflecting marker properties rather than true discrepancies with SNP-based estimates. In our cohort, infertile camels showed slightly reduced $H_{o}$ relative to fertile individuals, consistent with the well-documented link between greater autozygosity (including $F_{\mathrm{ROH}}$) and diminished fertility in cattle and horses [21,23].

GWAS was performed to identify genetic loci associated with fertility and coat color traits in the camel cohort. For the binary infertility trait, a case–control design compared allele frequencies between infertile (*n* = 5) and fertile camels (*n* = 91) using logistic regression models. Each SNP’s effect on log-odds of infertility was tested, yielding genome-wide *p*-values. No genomic regions were identified due to the low sample size in the infertility cases compared to the fertility control. To address this issue, we need to increase the sample size of infertile individuals and have a robust analysis. An even more powerful strategy would be using next-generation sequencing (NGS) with high coverage (35×) that can detect genomic regions associated with infertility (35). Additionally, GWAS for coat color (dark vs. light phenotypes) identified genome-wide significant associations on chromosome 3, with two SNPs (SEQ-RS1464642 and SEQ-RS1464884) showing *p*-values of 8 × $10^{-8}$ and 2 × $10^{-7}$, respectively. These SNPs, located at approximately 41.9 Mb, map near the *SLC30A5* gene, a well-characterized pigmentation locus in mammals. A recent study showed that *SLC45A2* is associated with white coat color, yellow eye color, and abnormal development in the hindlimb Alshanbari and Ibrahim [24]. Furthermore, reduction in *SLC30A2* results in a reduction in tyrosinase-related protein 1 (TYRP1) levels in melanocyte, resulting in a reduction in pigmentation (32).

Given our hypothesis that increased inbreeding reduces fertility in dromedary camels, it was essential to quantify genetic diversity and inbreeding at both the individual and breed levels. To elucidate the genetic architecture of the sampled dromedary camel populations, a suite of methods for assessing population structure and relatedness was employed.

Principal component analysis and ADMIXTURE analysis were used to discern patterns of genetic variation across the 96 camels sampled, which originated from seven recognized breeds (Asail, Saheli, Majaheem, Sofor, Shaele, Shageh, and Waddah). These analyses reduced the high-dimensional single-nucleotide polymorphism (SNP) dataset to a small number of components, thereby revealing breed differentiation and potential admixture. Identity-by-descent estimates identified close familial relationships (parent-offspring, full-sibling, and second-degree relationships) that may inflate the inbreeding coefficient and influence fertility. Our results showed that all five infertile individuals have higher inbreeding coefficients (F > 0.15). Furthermore, four females had high inbreeding coefficients and were sampled from the same herd. Our results support the research hypothesis, with some breeders confirming that they still use the same male for many years, making him mate with his offspring. Other samples were collected from normal animals. The results were consistent with similar studies that showed genetic diversity and high heterozygosity [3,23].

The lack of significant genomic regions associated with infertility in our GWAS analysis may be due to the polygenic nature of fertility traits or limited statistical power due to small sample size (*n* = 5 infertile individuals). The strong association between elevated inbreeding coefficients and infertility suggests that inbreeding depression affects multiple loci across the genome rather than single major effect genes.

## 5. Conclusions

In conclusion, our results demonstrate that inbreeding is associated with infertility in dromedary camels. Infertile individuals consistently showed F values >0.15, compared to <0.10 in fertile individuals. Moreover, our results demonstrated that the camel breeds under study exhibited moderate allelic diversity and low genetic differentiation, with most genetic variation residing within individuals rather than among breeds. The optimal number of ancestral populations is around six or seven, and the relatedness network identifies specific familial clusters. Inbreeding levels are generally low, although isolated individuals show higher inbreeding coefficients. Finally, genome-wide association analyses revealed a limited number of loci associated with coat color, suggesting a simple genetic architecture for this trait.

Further analysis is required to identify genomic regions associated with infertility in this species with larger sample sizes, which will improve our understanding and management of dromedary camel breeding systems. Implementation of breeding strategies that avoid mating closely related individuals should be considered to maintain reproductive performance in camel herds.

## Figures and Tables

**Table 1:** Summary of dromedary camel breeds used in this study with phenotypic descriptions.

Breed	*n*	Coat Color	Body Size	Purpose	Region
Asail	2	Brown	Small	Racing	Coast
Saheli	5	Brown body with dark brown to black wool	Small to medium	Meat/milk	West coast
Majaheem	17	Black	Large	Meat/milk	Desert
Sofor	12	Dark brown with black wool	Medium to large	Meat/milk	Desert
Shaele	14	Brown	Medium to large	Meat/milk	Desert
Shageh	18	Light brown	Medium to large	Meat/milk	Desert
Waddah	28	White	Medium to large	Meat/milk	Desert

**Table 2:** Sex distribution of dromedary camels by breed.

Breed	Male	Female	Total
Asail	2	0	2
Saheli	0	5	5
Majaheem	2	15	17
Sofor	4	8	12
Shaele	6	8	14
Shageh	3	15	18
Waddah	3	25	28
**Total**	**20**	**76**	**96**

## Data Availability

The datasets generated and analyzed during the current study are available from the corresponding author on reasonable request. SNP genotyping data will be deposited in a publicly accessible repository upon publication. All camel owners provided informed consent, and procedures were approved by the Qassim University Animal Ethics Committee.

## Abbreviations

The following abbreviations are used in this manuscript:

AMOVA	Analysis of Molecular Variance
F	Inbreeding coefficient
FST	Fixation index
GWAS	Genome-wide association study
IBD	Identity-by-descent
PCA	Principal component analysis
ROH	Runs of homozygosity
SNP	Single-nucleotide polymorphism
